# Chronic calcium pyrophosphate crystal inflammatory arthritis induced by extreme hypomagnesemia in short bowel syndrome

**DOI:** 10.1186/1471-230X-12-129

**Published:** 2012-09-22

**Authors:** Markus Hahn, Martin Raithel, Alexander Hagel, Teresa Biermann, Bernhard Manger

**Affiliations:** 1 Department of Medicine I, Gastroenterology, University Erlangen-Nürnberg, Ulmenweg 18, Erlangen, 91054, Germany; 2 Department of Medicine III, Rheumatology, University Erlangen-Nürnberg, Ulmenweg 18, Erlangen, 91054, Germany; 3 Department of Psychiatry and Psychotherapy, University Erlangen-Nürnberg, Schwabachanlage 6, Erlangen, 91054, Germany

**Keywords:** SBS, Hypomagnesemia, Chondrocalcinosis, Pseudogout, CPPD

## Abstract

**Background:**

Short bowel syndrome (SBS) may induce a plethora of clinical symptoms ranging from underweight to nutrient-, vitamin- and electrolyte deficiencies. The objective of this case report is to illustrate how demanding the management of a 60 year old patient with SBS and recurrent joint attacks was for different medical disciplines.

**Case presentation:**

The patient with SBS presented with a body mass index of 16.5 kg/m^2^ after partial jejunoileal resection of the small intestine with a six year long history of recurrent pain attacks in multiple peripheral joints, chronic diarrhoea and food intolerances. Pain attacks occurred 4–5 times a week with a median consumption of 15 mg prednisone per day. The interdisciplinary workup after several gastroenterologic, rheumatologic, radiologic, psychiatric and orthopedic consultations is shown including successful treatment steps.

Clinical diagnosis revealed no systemic inflammatory disease, but confirmed extreme hypomagnesemia (0.2 mmol/l) after reproducible pathological magnesium resorption tests as causative for chronic calcium pyrophosphate crystal inflammatory arthritis (pseudogout, chondrocalcinosis).

Multidisciplinary treatment included application of colchicines, parenteral nutrition and magnesium substitution, antiperistaltic agents and avoidance of intolerant foods. Normalization of magnesium levels and a marked remission of joint attacks were achieved after six months with significant reduction of prednisone to 1.5 mg/day.

**Conclusion:**

Despite the rarity of this condition, it is important to know that hypomagnesaemia may be associated with calcium pyrophosphate crystal inflammatory arthritis (chondrocalcinosis) and that SBS patients may be prone to develop extreme hypomagnesaemia causing recurrent joint attacks without systemic inflammation.

## Background

Symptoms of short bowel syndrome (SBS) with mild, moderate or severe intestinal failure and/ or associated metabolic complications are estimated to occur if less than 200 cm of small bowel have been retained [1-3]. It has been reported to show a wide spectrum of potential complications like underweight, food intolerance, osteoporosis, steatorrhea, chologenic diarrhea with loss of bile salts, electrolytes and vitamins as well as megaloblastic anemia or renal calcium oxalate stone formation etc. [1-3]. Although SBS patients are often primarily referred to gastroenterological or nutritional hospitals, several other medical disciplines may be involved because of extraintestinal affections or complications like hormonal dysbalances, neurological symptoms (polyneuropathy), psychiatric or psychosocial co-morbidities (anxiety, chronic fatigue and depression) or orthopedic complications (osteoporosis) etc. Thus, often only an interdisciplinary approach may be helpful to manage patients with SBS, and at least, each patient has to be treated individually, based on more or less conserved intestinal functions or manifested deficiencies [1-3]. The present case demonstrates how difficult clinical challenges can be in SBS and shows how intestinal resection may lead to severe metabolic consequences, recurrent joint attacks and food intolerances.

### Case presentation

A 60 year old man presented with a 30 year history of short bowel syndrome (SBS) caused by jejunoileal resection of 2/3 of the ileum in 1980 after abdominal trauma. The bodyweight was very low (53 kg) with a body mass index (BMI) of 16.5 kg/m^2^. After abdominal resection he had had intravenous nutrition for some years in the 1980s, but this could be finished after successful intestinal adaptation.

At presentation in 2010, the patient complained primarily of recurrent episodes of joint pain predominantly in shoulders, knees, and ankles. He took 10 – 20 mg prednisone per day and sometimes a non-steroidal analgesic to cope with the articular pain. Six years ago he had experienced an episode of severe arthritis of the knees rendering him immobile. Arthroscopy at that time revealed highly active synovitis. Back then the patient suffered from progressive dysthymia, slept long into the day, developed reduced activity and self-confidence. He complained of anxiety, tremor, restlessness and impaired concentration and was diagnosed among others by Hamilton Depression Rating Scale as having a major depressive episode [4,5]. These neuro-psychiatric symptoms had been resistant to treatment with duloxetine, amitriptyline and pregabaline for nearly two years.

Further on he reported significant underweight (BMI = 16.5 kg/m^2^), muscular weakness, 3–4 pasty stools a day and suffered from recurrent abdominal discomfort, distension and pain. He had further significantly restricted oral nutrition because of malabsorption for some foodstuffs, lactose, fat, vitamin- and electrolyte supplements. His physical and psychological efficiency was considerably reduced.

### Diagnostic and therapeutic procedures

Physical examination yielded signs of malnutrition, sarcopenia and underweight. Otherwise there were no irregularities. Electrocardiography was normal without signs of arrhythmia or elongation of intervals.

Further additional normal diagnostic tests included:

• Serum electrophoresis

• Total serum IgE and food specific IgE

• Folic acid and vitamin B6

• Anti nuclear antibodies

• Deoxyribonucleic acid antibodies

• Immune complexes

• Ferritin

• Transabdominal sonography

Systemic and gastrointestinal symptoms were assessed by the Erlangen Score for food intolerances and it showed a moderate to severe clinical disease activity with 25 points (normal < 5, Table 1) [6]. The patient complained about pain upon active and passive motion of both knees and ankles.

**Table 1:** Clinical parameters and score activities of the patient

**Parameter**	**Before treatment**	**6 months after treatment**
Body weight (kg)	52	59
Body mass index (kg/m^2^)	16.4	18.0
Stool frequency (stools/day)	4-6	1-2
Disease activity by food intolerance score (n = points)	25	5
Disease activity (joint attacks/week)	4-5	none
Consumption of prednisone (mg/day)	15	1.5
Number of painful joints	4	0
Quality of life	Severe reduction	Slight reduction

Clinical parameters and score activities of the patient with SBS, extreme hypomagnesemia and calcium pyrophosphate crystal inflammatory arthritis (chondrocalcinosis) at the time of diagnosis and 6 months after intense interdisciplinary treatment.

While blood count and inflammatory parameters were inconspicuous, serum levels of Mg were extremely low (0.2 mmol/l; n 0.7-1.1) in repeated tests, causing symptoms of muscle weakness, restlessness and paraesthesias. Blood calcium (1.7 mmol/l; n 2.02-2.60 mmol/l) as well as vitamin D (12 ng/ml, n 16–70 ng/l) were also depleted with clinically manifest osteoporosis and multiple fractures of vertebrae. Further supplementary laboratory data is shown in Table 2 with low serum protein levels (58.3 g/l; n 61–81 g/l) and a reduced zinc level (64 μg/dl; n 70–120 μg/dl).

**Table 2:** Laboratory data for the patient

**Parameter**	**Before treatment**	**6 months after treatment**
Hemoglobin (12 – 16 g/dl)	11.8	13.1
Leucocytes (4 – 10000/μl)	8600	7100
Thrombocytes (140 – 400000 μl)	354000	295000
ESR	7/9	4/6
CRP mg/l (<5)	0.4	1.1
Proteins g/l (66–83)	58.3	66.1
Albumin g/l (35–55)	41.1	39.4
Sodium mmol/l (135–145)	138	142
Potassium mmol/l (3,6-4,8)	3.4	4.1
Magnesium, mmol/l (0.7–1.1)	0.2	0.6
Calcium, mmol/l (2.1–2.7)	1.7	2.2
Phosporus mg/dl (2.5-4.5)	3.6	3.5
Transferrin saturation,% (16-45%)	37.5	36.4
Uric acid mg/dl (3.4-7)	5.8	5.8
Serum creatinine mg/dl (0.84-1.25)	1.1	1.06
Urea mg/dl (17–43)	51	58
Zinc, μg/dl (72–175)	64	89
Triglycerides mg/dl (<200)	312	171
Cholesterol mg/dl (<200)	143	147
25OH vitamin D, ng/ml (30–70)	12	38
Vitamin B12 (200 – 1100 pg/ml)	151	241
Parathormone pg/ml (10–65)	11.3	15

Laboratory data for the patient with SBS, extreme hypomagnesemia and calcium pyrophosphate crystal inflammatory arthritis (chondrocalcinosis) at the time of diagnosis and 6 months after intense interdisciplinary treatment.

Uric acid was normal (5.8 mg/dl). Renal loss of Mg was ruled out through normal values of daily urinary Mg excretion and fractional clearance of Mg. To prove malabsorption as the origin of Mg depletion, a Mg resorption test was performed twice with 750 mg magnesium oxide (18.4 mmol Mg, Magnetrans forte 150 mg, Stada GMBH, Bad Vilbel, Germany). At the time point 0 minutes the patient took 5 tablets with 250 ml water and at the time points 60, 120 and 240 minutes the serum magnesium levels were determined. No uptake could be observed during the period of 240 minutes at a serum level of 0.2-0.3 mmol/l (n 0.7-1.1). Hypomagnesemia as an adverse side effect of pharmacologic treatment was not found, as the patient had not taken any drugs known to affect Mg-homeostasis. Hemochromatosis and hyperparathyroidism as a potential reason for CPPD deposition were ruled out by normal values of ferritin, transferrin saturation and parathyroid hormone (Table 2).

Furthermore, bacterial colonization of the remnant parts of the ileum could be excluded through a hydrogen breath test with normal fasting H_2_ concentrations and no premature increase of H_2_ gas exhalation.

Functional testing of blood leucocytes showed clearly increased leukotriene production in response to acetylsalicylic acid, characteristic of NSAID (non steroidal anti inflammatory drugs) intolerance [6,7]. This finding may explain some types of food intolerances reported from the patient which contain salicylates, but also benzoic acid, tartrazine etc. and may be causative for intolerance of some vitamin and electrolyte supplements [6,8].

Endoscopic-histologic investigation by double balloon enteroscopy until 135 cm aborally showed no inflammatory, collagenous or lymphocytic lesions in the duodenum, jejunum or ileum. No villous atrophy was found and only discrete mild inflammatory infiltrates were focally seen within the small bowel. From enteroscopic and radiologic evaluation the length of the remaining small bowel was estimated to be around 185 – 195 cm. Subtle atrophy and a slightly spotted erythema were discovered in the antrum of the stomach.

**Figure 1:**
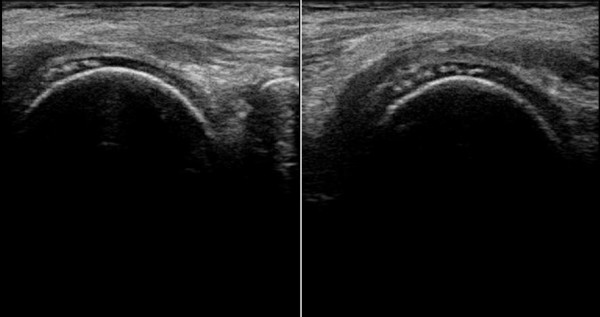
Sonographic detection of CPPD inflammatory arthritis (chondrocalcinosis) in the femoral condyles of both knees in SBS with extreme hypomagnesemia.

**Figure 2:**
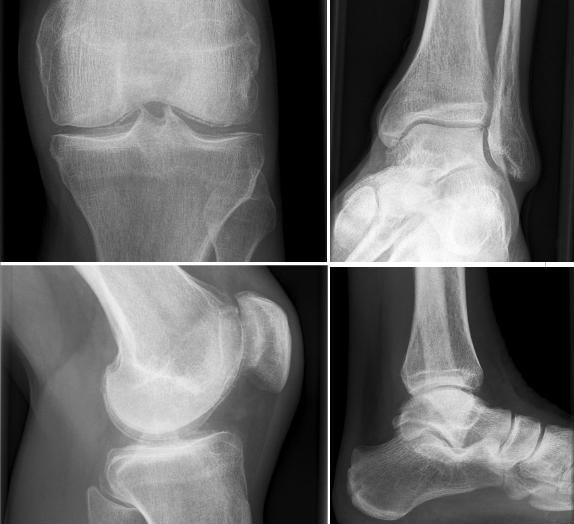
Radiologic findings of CPPD inflammatory arthritis (chondrocalcinosis) with tiny calcification of menisci and of hyaline cartilage in knees and ankles in SBS with extreme hypomagnesemia.

Rheumatologic examination revealed pain but no actual swelling in knees and ankles. Sonographic imaging showed linear calcification within the hyaline joint cartilage in both knees (see Figure 1). Radiographic results also confirmed the presence of symmetrical chondrocalcinosis in knees and ankles characteristic for CPPD deposition (see Figure 2). Aspiration of synovial fluid could not be performed in absence of effusions.

After exclusion of other inflammatory or autoimmune diseases and in view of the above mentioned clinical and radiological signs, oligoarticular chronic CPPD crystal inflammatory arthritis due to hypomagnesemia induced by SBS was diagnosed.

### Interdisciplinary treatment regimen significantly improved disease course

An interdisciplinary regimen based on the following principles was established:

1. According to the EULAR recommendations [9] for the management of chronic CPPD crystal inflammatory arthritis a therapy with low-dose colchicine (1 mg/d) and prednisone (5 mg/d) was established. Due to the diagnosed non-steroidal drug intolerance, this group of analgesics was no longer applied [6-8]. For occasional periods of analgetic treatment, tramadol or metamizol were taken.

2. Parenteral supplementary nutrition was administered monthly for six months with regular infusions of trace elements, electrolytes, Mg, vitamin B12, glutamine and lipids (For trends of laboratory data see Table 2).

3. Further nutritional advice was given to the patient to improve oral nutrition. He was especially taught to consume high caloric liquid foods, foods rich in Mg, lipids (mostly medium-chain triglycerides), vitamin D and proteins (e.g. curds with fat content of 20 – 40%).

4. Further oral trace elements, folic acid and Tinctura opii normata (containing 0.9-1.05% morphine, 0.1% codeine and 0.3% thebaine (Maros GmbH, Fürth, Germany) as an antiperistaltic agent were administered orally. Pancreatic enzymes were prescribed to increase digestive capacity of the gastrointestinal tract, as underweight, multiple food intolerances with accelerated intestinal transit time and fat intolerance were present along with pasty stools. Accelerated transit time was suspected as one cause to reduce normal pancreatic digestion of foods, resulting in fat intolerance and abdominal discomfort.

5. According to the known intolerances, foodstuffs containing lactose or salicylates had to be excluded from the schedule [6,7] and lactase supplementation was implemented.

6. The psychiatric condition was treated with a medication regime comprising duloxetine, low dose amitriptyline and also intermittently pregabaline in various dosages as well as supportive psychotherapy.

Over the course of treatment blood levels of Mg normalized within six months (Table 2). The rate of joint attacks decreased remarkably from 4–5 times a week to approximately only one attack per month, leading to an improved quality of life which enabled the patient to get back into work. After one year of treatment the prednisone dose could be reduced to 1.5 mg per day. Interestingly, the above mentioned neuro-psychiatric symptoms as well as sleep disturbances resolved after a few weeks of magnesium and nutrients supplementation while applying an interdisciplinary treatment regimen, but only a moderate effect on the perception of pain could be accomplished. With the gradual improvement of the patients’ mind, resolution of depressive symptoms and increase of daily activities, several psychiatric consultations confirmed remission of the depressive episode. Thus, initially required drugs like duloxetine, amitryptiline etc. could be finished in the long-term follow-up and today the patient only uses benzodiazepine on demand in the case of problems getting to sleep.

## Discussion

Patients may develop a plethora of clinical symptoms in SBS, when their remaining length of functional gut is insufficient for adequate absorption. Therefore, a supplementation of nutrients, water and electrolytes is usually required to maintain health and growth, respectively [10]. This case report describes a patient with extreme hypomagnesaemia and consecutive chronic CPPD crystal inflammatory arthritis due to a long history of SBS, which was inadequately supplemented and treated over years. Despite arthroscopy six years before CPPD deposition remained unrecognized. In the end, multiple current diagnostic procedures, including magnesium resorption test, revealed hypomagnesaemia as the most likely explanation for CPPD deposition. Other associated disorders such as hyperparathyreoidism, renal Mg loss and hemochromatosis were ruled out.

**Figure 3:**
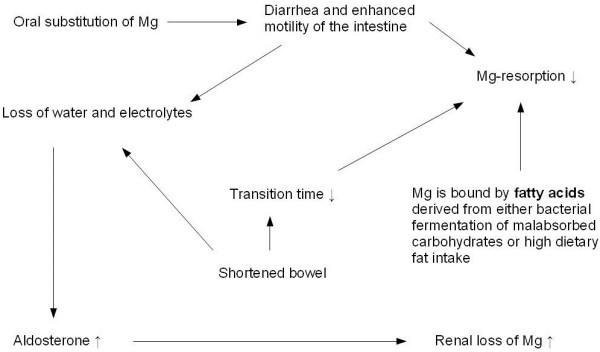
Pathophysiology of hypomagnesaemia in SBS.

Since Mg-absorption testing demonstrated no resorption of Mg due to shortened small intestinal length with increased peristalsis because of lactose, fat and NSAID intolerance, the malabsorptive state and the SBS were considered as the reason for this extreme Mg deficiency. Hypomagnesaemia is a common feature of SBS. Fatty acids derived from either bacterial fermentation of malabsorbed carbohydrates or high dietary fat intake (long-chain triglycerides) bind Mg and prevent absorption [10]. Another contributing factor for Mg deficiency in SBS is hyperaldosteronism induced by loss of water and salt which increases renal wasting of Mg [11]. In addition, oral supplementation of Mg is known to cause intestinal motility and diarrhea, which can further compromise adequate Mg absorption. Finally, our patient had reduced 1,25-hydroxy-vitamin D levels, which further hampers normal jejunal Mg absorption. Thus, our patient had several pathophysiologic mechanisms contributing to hypomagnesaemia (See Figure 3 for illustration). Therefore, parenteral substitution of Mg along with other nutrients was required to assure a sufficient Mg supply [10].

Mg is a divalent cation mostly located intracellularly. It plays an important role in enzymatic processes. In healthy individuals homeostasis of Mg is regulated effectively, as sufficient mechanisms in the gastrointestinal tract and the kidney exist. However, if these mechanisms are affected in underlying disease, Mg depletion can cause a wide range of symptoms such as neuromuscular manifestations (muscular cramps, vertigo, tetany), cardiac and vascular manifestations (arrhythmias, hypertension), metabolic sequels and psychiatric manifestations (depression, fatigue) [11,12].

As seen in this case, severe Mg depletion resulted in CPPD deposition with clinical manifestations of chronic inflammatory arthritis [13-16] and was responsible for muscular weakness, fatigue and the depressive episode.

Chondrocalcinosis, is defined as the deposition of calcium pyrophosphate dihydrate in hyaline or fibrous cartilage [14]. In radiography delicate linear calcification of the cartilage can be seen. The prevalence of chondrocalcinosis increases with age (10-15% for people between 65 and 75 years) and is hence called sporadic in patients older than 60 years, whereas in younger individuals there are several putative underlying disorders causing CPPD deposition disease, such as hemochromatosis, hyperparathyroidism, hypomagnesemia or hypophosphatemia [14]. Although often asymptomatic, CPPD crystals can cause inflammation of joints resulting in attacks of pseudogout [17]. Typical joints affected by CPPD-induced arthritis are knees and ankles as demonstrated in our patient [14]. The association between low levels of Mg and CPPD deposition is not fully understood. As Mg acts as a cofactor for various phosphatases, a deficiency can lead to higher amounts of pyrophosphate, a necessary precursor for the formation of CPPD crystals [18].

The linkage between SBS and Mg depletion on the one hand, as well as between Mg depletion and CPPD deposition disease on the other hand, is known. Whereas Mg deficiency causing CPPD deposition is due to renal failure in most cases, such as in Bartter's or Gitelmann's syndrome [13,18,19], the connection of SBS causing extreme hypomagnesemia with subsequent CPPD deposition is scarce. To our knowledge, there have been only three cases described with a definite diagnosis of CPPD-induced arthritis (chondrocalcinosis) due to SBS [20].

## Conclusions

Despite the rarity of this condition, it is important to know that SBS can be connected to CPPD deposition disease (chondrocalcinosis), presenting with acute or chronic arthritis and therefore SBS patients should be thoroughly investigated and, if necessary, receive parenteral treatment for Mg deficiency. Colchicine presents a favorable long-term medication with steroid-sparing effect, especially in the initial period of treatment [9]. Combined with several other therapeutic steps, intestinal Mg resorption rate was also improved by treating associated food intolerances, administering opioids to reduce bowel transit time and correcting vitamin D deficiency.

### Consent

Written informed consent was obtained from the patient for publication of this Case report and any accompanying images. A copy of the written consent is available for review by the Series Editor of this journal.

## Abbreviations

SBS: Short bowel syndrome; CPPD: Calcium pyrophosphate dihydrate; Mg: Magnesium; NSAID: Non steroidal anti inflammatory drugs; BMI: Body mass index.

## Competing interests

The authors declare that they have no competing interests.

## Authors' contributions

Each author made substantial contributions to conception and design (MR, BM, TB, and MH), and/or acquisition of data (MR, MH, and AH), and/or analysis and interpretation of data (MH, AH, MR). All Authors participated in drafting the article or revising it critically for important intellectual content and all authors give final approval of the version to be published.

## Pre-publication history

The pre-publication history for this paper can be accessed here:

http://www.biomedcentral.com/1471-230X/12/129/prepub
